# The effect of cyclodextrin complexation on the solubility and photostability of nerolidol as pure compound and as main constituent of cabreuva essential oil

**DOI:** 10.3762/bjoc.13.84

**Published:** 2017-05-05

**Authors:** Joyce Azzi, Pierre-Edouard Danjou, David Landy, Steven Ruellan, Lizette Auezova, Hélène Greige-Gerges, Sophie Fourmentin

**Affiliations:** 1 Bioactive Molecules Research Group, Doctoral School of Sciences and Technologies, Faculty of Sciences, Jdaidet El-Matn, Lebanese University, Lebanon; 2 Unité de Chimie Environnementale et Interactions sur le Vivant (UCEIV, EA 4492), SFR Condorcet FR CNRS 3417, ULCO, F-59140 Dunkerque, France

**Keywords:** cabreuva essential oil, cyclodextrins, nerolidol, photostability, solubility

## Abstract

Nerolidol (Ner), a major component of many plant essential oils, is known for its various biological properties. However, the low solubility of Ner in water and its susceptibility to degradation limit its application. The aim of our study was to improve the solubility and photostability of Ner through its encapsulation in different cyclodextrins (CDs). The formation constants of *cis*-, *trans*-Ner and their commercial mixture with various CDs (α-CD, β-CD, γ-CD, HP-β-CD, RAMEB, CRYSMEB and SBE-β-CD) were determined by phase solubility studies and confirmed by the spectral displacement UV-visible method. The solubility of cabreuva essential oil (EO) rich in *trans*-Ner was also evaluated by total organic carbon (TOC) analysis. The encapsulation efficiency (EE %) of Ner in HP-β-CD solid complexes was assessed by HPLC. The structural characterization of CD/*trans*-Ner inclusion complex was then conducted by NMR spectroscopy followed by molecular modelling studies. The effect of encapsulation on the Ner photostability was also carried out over time under UVB irradiation. A_L_-type phase-solubility diagrams were obtained, suggesting the formation of 1:1 CD/Ner inclusion complexes. The solubility of Ner was enhanced by approximately 70-fold in the presence of 10 mM HP-β-CD. Moreover, high EE % values were obtained for 5:1 and 10:1 HP-β-CD:Ner molar ratios. NMR and molecular modelling studies revealed the most stable structure for *trans*-Ner inside the CD cavity with the OH group oriented towards the wider rim of the CD. Finally, CD encapsulation of Ner as pure compound or as main component of the cabreuva EO, protected it from degradation. This effect was more pronounced as the concentration of CD increased. These findings suggested that CDs are promising encapsulating carriers for Ner by enhancing its solubility and stability and thereby its application in food industry.

## Introduction

**Figure 1:**
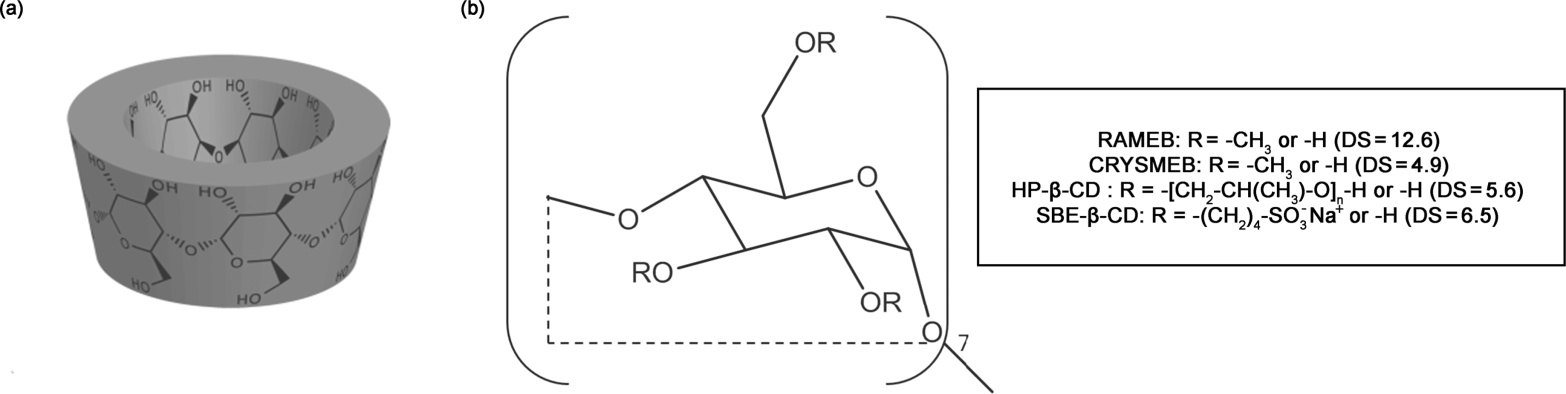
Chemical structure of β-CD (a) and β-CD derivatives (b).

Nerolidol (Ner, 3,7,11-trimethyl-1,6,10-dodecatrien-3-ol), an acyclic sesquiterpene obtained from fresh flowers of bitter orange and found in many other plants [1], is extensively used in perfumery. It was also approved by the U.S. Food and Drug Administration as a food flavoring agent and included by the Council of Europe in the list of substances granted A [2]. In nature, Ner occurs as a mixture of two isomers: *cis* and *trans*, with *trans* being the most abundant. The two isomers are distributed differently in various plant parts and among species. *trans*-Ner is present in high percentage (58–80%) in cabreuva essential oil (EO) (*Myrocarpus fastigiatus*) [3]. Due to its antimicrobial effects against a wide range of microorganisms such as *Staphylococcus aureus*, *Salmonella enterica* and *Aspergillus niger* [4], Ner can be employed as natural alternative to traditional synthetic food preservatives. To achieve this goal, Ner should be dissolved partially or totally in aqueous media to provide an efficient interaction with the pathogens [5]. However, Ner is poorly soluble in water and is also subject to degradation during storage. Nanoencapsulation techniques may improve Ner solubility and stability allowing to produce functional foods with enhanced functionality and stability [6]. Various encapsulation materials are used for food applications such as liposomes, phytosomes, cyclodextrins, dendrimers and many others [7–9]. The use of cyclodextrins (CDs) in food industry is currently flourishing due to their high potential to solubilize, stabilize and protect flavors and food ingredients [10–13]. CDs are non-toxic water-soluble cyclic oligosaccharides composed of six, seven or eight α-(1→4) glucopyranose units giving rise, respectively, to α-, β- and γ-CDs [14]. Their most notable characteristic is their ability to form inclusion complexes with poorly soluble guest molecules which are entrapped in the hydrophobic cavity of CDs. Chemical modifications of the hydroxy groups of CDs can be performed to obtain water-soluble CD derivatives like hydroxypropyl-β-CD (HP-β-CD), methylated CDs (randomly methylated β-CD, RAMEB, or a low 2-O-methylated-β-CD, CRYSMEB) and sulfobutylether-β-CD (SBE-β-CD) (Figure 1). Molecular complexation in CDs has been widely proposed for EOs to limit their degradation during processing and storage and to offer a good dispersion of compounds in the aqueous media thus enhancing their biological properties [15–16].

To the best of our knowledge, no literature data reported the encapsulation of Ner in CDs. In this study, for the first time, CD/Ner inclusion complexes were characterized in solution and in solid state. Formation constants (*K*_f_) were determined for Ner with seven CDs: α-CD, β-CD, γ-CD, HP-β-CD, RAMEB, CRYSMEB and SBE-β-CD using phase solubility studies coupled to HPLC. The *K*_f_ values were determined for the separate isomers and their commercial mixture. To confirm the *K*_f_ values obtained for the isomeric mixture, UV-visible spectroscopy was employed using a competition method. The solubility of cabreuva EO rich in *trans*-Ner was also assessed with HP-β-CD using the total organic carbon method developed in our laboratory [17]. In addition, ^1^H and 2D ROESY NMR experiments and molecular modelling were performed to investigate the orientations of *trans*-Ner inside the CD cavity. Next, the effect of encapsulation on the Ner photodegradation rate under UVB irradiation was examined. Solid HP-β-CD/Ner complexes were also prepared and the encapsulation efficiency (EE %) was determined.

## Results and Discussion

### Phase solubility studies

**Figure 2:**
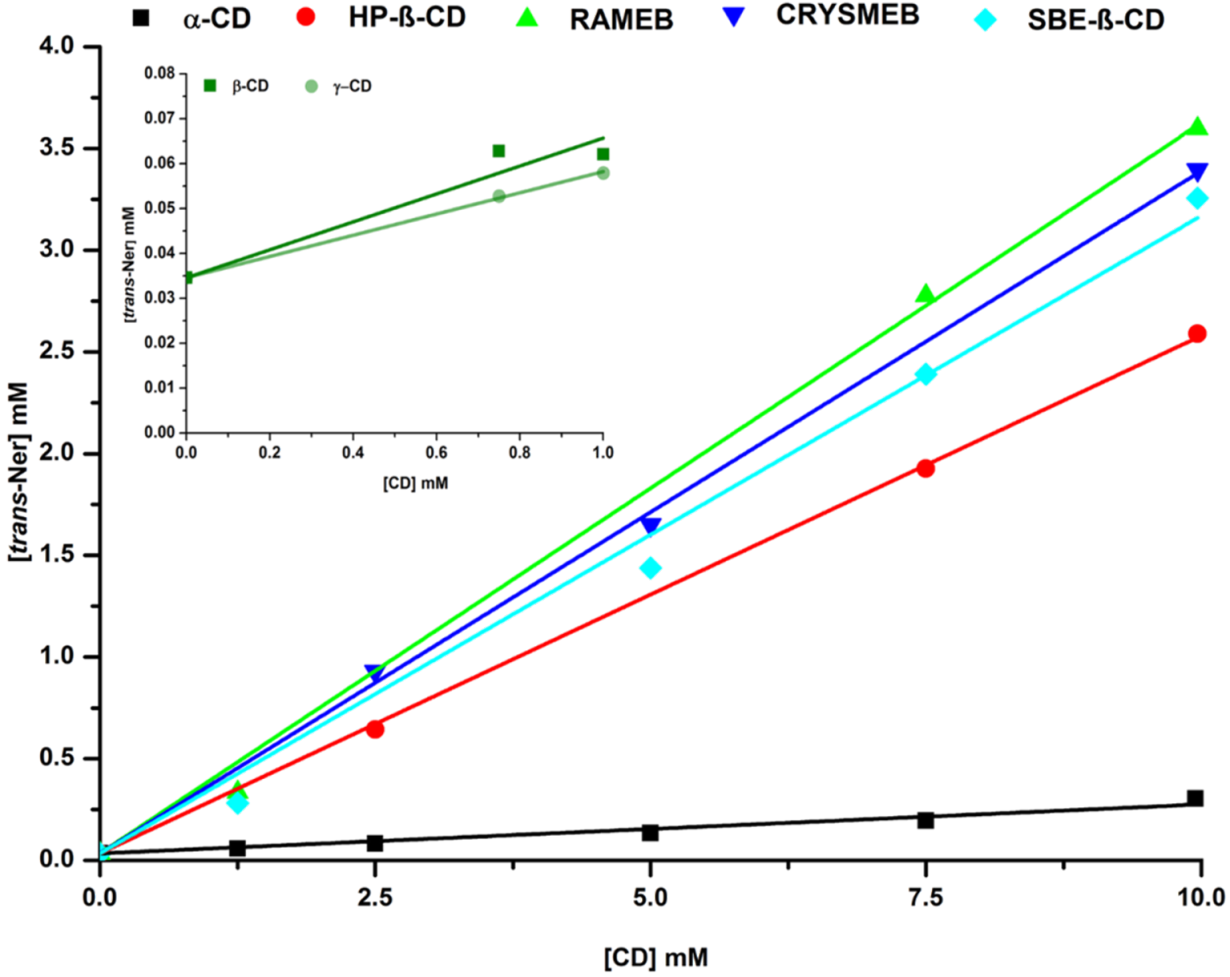
Phase solubility diagrams of CD/*trans*-Ner inclusion complexes.

Considering the low aqueous solubility of Ner, phase-solubility studies were conducted to examine the ability of different CDs (α-CD, β-CD, γ-CD, HP-β-CD, RAMEB, CRYSMEB and SBE-β-CD) to solubilize Ner. All concentrations were determined for each isomer from their commercial mixture by HPLC. The mean intrinsic solubility (S_0_) of *cis*- and *trans*-Ner in water at room temperature was estimated to be about 5 and 7 µg/mL, respectively. The phase-solubility diagrams showed that the solubility of both isomers increased linearly with CD concentration. Thus, an about 60- and 80-fold increase in solubility is achieved, in the presence of 10 mM HP-β-CD, for *cis*- and *trans*-Ner (284 µg/mL and 578 µg/mL, respectively). Moreover, the curves obtained for all CDs/Ner inclusion complexes followed an A_L_-type profile except for β and γ-CD where B_s_-type profiles were obtained (Figure 2). For A_L_-type phase solubility diagrams, the slope was less than unity which indicates the formation of 1:1 inclusion complexes [18]. The *K*_f_ values (Table 1) were then calculated as indicated in Equation 1 (experimental part). For β-CD and γ-CD, precipitation occurred at concentrations above 1 mM.

**Table 1:** Formation constants (*K*_f_), solubility enhancement ratio (S_t_/S_0_) (in the presence of 10 mM CD, except for β-CD and γ-CD, 1mM) and complexation efficiency (CE) of *cis* and *trans*-Ner with different CDs.

	cis-Ner			trans-Ner			Ner^b^
CDs	*K*_f_ (M^−1^)	S_t_/S_0_	CE^a^	*K*_f_ (M^−1^)	S_t_/S_0_	CE	*K*_f_ (M^−1^)

α-CD	81	1.85	0.002	619	8.80	0.021	418
β-CD	769	1.49	0.019	1233	1.80	0.043	1238
γ-CD	1866	2.76	0.046	752	1.68	0.026	1435
HP-β-CD	5540	51.53	0.137	9515	75.37	0.328	10365
RAMEB	8149	72.62	0.202	15577	104.40	0.537	19450
CRYSMEB	8787	72.77	0.217	14036	98.80	0.484	15412
SBE-β-CD	9240	80.20	0.229	13264	94.66	0.457	17768

^a^CE: complexation efficiency ([CD/Ner])/[CD]). ^b^Ner: formation constant for the commercial mixture.

We can notice from Table 1, that the *K*_f_ values of CD/*cis*-Ner were generally lower than those of CD/*trans*-Ner. This is evidently attributed to the different spatial orientations of the isomers: *trans*-Ner adopts a linear conformation while *cis*-Ner has a more twisted one. Consequently, the interaction with the CD cavity is more favorable for the *trans*-Ner isomer leading to a more stable inclusion complex [16]. An exception is observed for γ-CD, where *cis*-Ner is better encapsulated than *trans*-Ner, owing to the larger cavity of this CD.

Phase-solubility studies also allow the determination of the complexation efficiency (CE) permitting to evaluate the solubilizing potential of CDs. CE is obtained either from the slope of phase solubility curve or by dividing the concentration of CD in its complexed form ([CD/Ner]) by its concentration in its free form ([CD]) (Equation 2) [19]. For both isomers, the CE values of CD derivatives were higher than those of native CDs. For instance, the CE values of *trans*-Ner for HP-β-CD, RAMEB and SBE-β-CD were, respectively, 0.328, 0.537 and 0.457 versus 0.043 for β-CD (Table 1). These results showed that RAMEB is the best solubilizer among the studied CDs. The solubility enhancement of this CD towards many compounds has been reported in the literature [20–22]. However, HP-β-CD is the only β-CD derivative cited in the FDA’s list of Inactive Pharmaceutical Ingredients among the studied derivatives, therefore, further studies will focus on this CD.

### UV–visible spectroscopy

Formation constants between Ner (mixture of *cis* and *trans* isomers) and HP-β-CD was also evaluated by a UV-visible competition method with methyl orange (MO) as a competitor [23]. Before starting the competition experiment, the *K*_f_ value of the HP-β-CD/MO inclusion complex was determined by direct titration; the data were consistent with those reported in the literature [24]. The competition method measures the spectral variation of MO upon the addition of Ner to a solution containing HP-β-CD and MO. The addition of Ner induced an increase in MO absorbance, which indicates the formation of inclusion complex between Ner and HP-β-CD. The obtained spectral variations revealed once again the formation of 1:1 inclusion complexes (data not shown). The *K*_f_ values of HP-β-CD/Ner inclusion complexes (Table 2) were in good agreement with the phase solubility results. However, a slight increase was observed in *K*_f_ values obtained from the phase solubility studies. Indeed, formation constants determined by this method are generally overestimated since several effects are combined and not only complexation [25].

**Table 2:** Formation constants (*K*_f_) of HP-β-CD inclusion complexes with Ner obtained by phase solubility and UV-visible competition method.

Formation constants (M^−1^)		HP-β-CD

Phase solubility	Ner	10365
UV–visible	MO	7429
	Ner	8168

### Phase solubility studies of cabreuva essential oil

**Figure 3:**
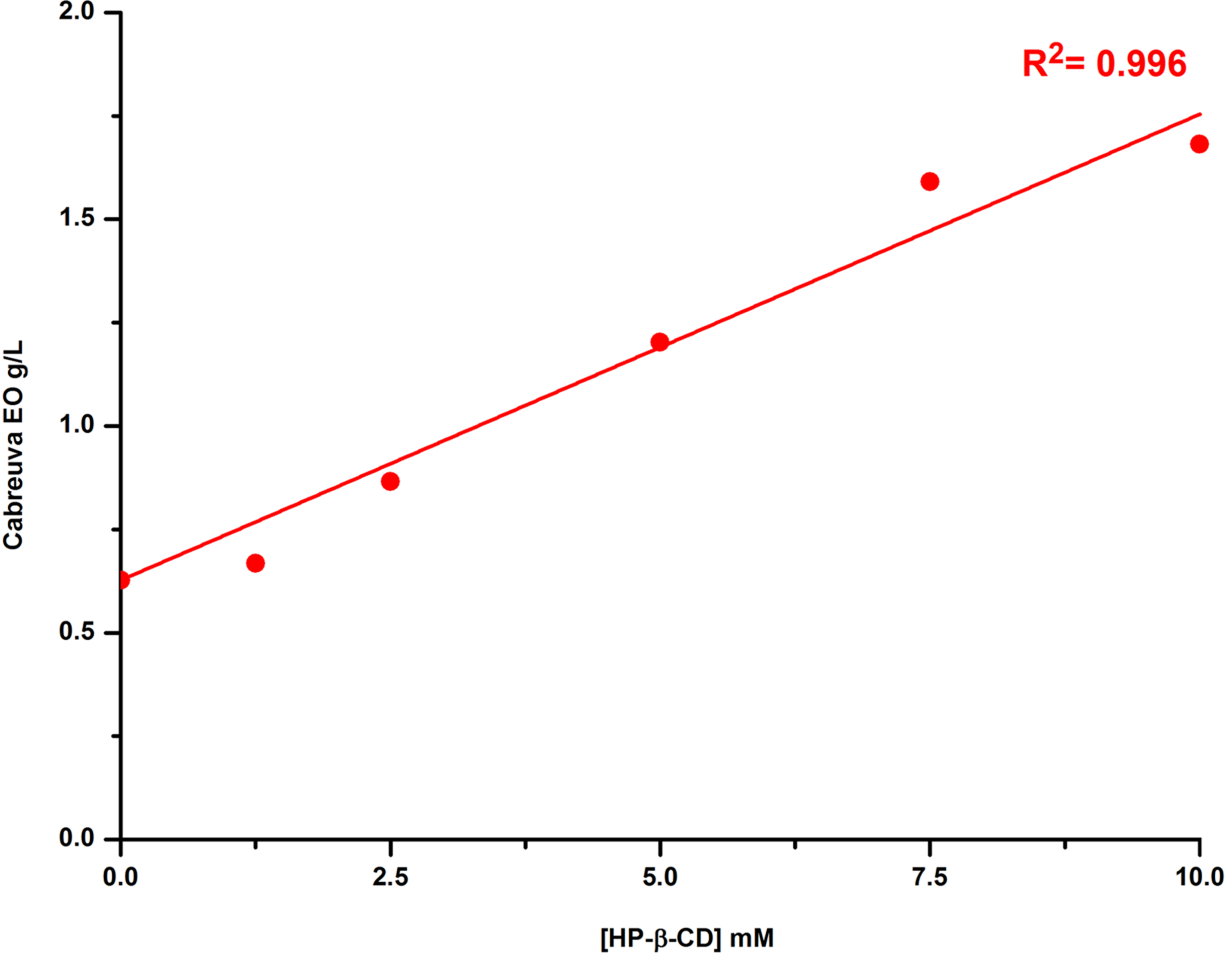
Phase solubility profile of cabreuva EO obtained by the TOC method.

Phase solubility studies were also performed for the cabreuva EO, an EO rich in *trans*-Ner (64.54%), at different HP-β-CD concentrations (0, 1.25, 2.5, 5, 7.5 and 10 mM). The phase solubility profile was obtained by the Total Organic Carbon (TOC) method [17]. The mass concentration of EO (g/L) obtained from the EO standard calibration curves, was plotted against CD concentration (Figure 3). As illustrated in Figure 3, the intrinsic solubility of EO was found to be of about 0.63 g/L. A linear increase in EO solubility was observed with HP-β-CD concentration (R^2^ = 0.996). The solubility of cabreuva EO increased from 0.63 to 1.7 g/L in the presence of 10 mM HP-β-CD (≈3-fold increase). These results showed that HP-β-CD is able to enhance the aqueous solubility of cabreuva EO. Other studies have also demonstrated that CD could significantly increase the solubility of EO components [26].

### Characterization of solid inclusion complexes

HP-β-CD/Ner mixture was prepared as a solid inclusion complex by the freeze-drying method in the presence of 50 and 100 mM CD and a fixed Ner concentration (10 mM). The encapsulation efficiency (EE %) of the Ner mixture in CD at 5:1 and 10:1 CD/Ner molar ratios was evaluated by HPLC. A high EE % was obtained for both HP-β-CD/Ner molar ratios (99 and 100% for 5:1 and 10:1, respectively) in good agreement with phase solubility results. The Ner contents were equal to 0.032 and 0.020 mg_Ner_/mg_solid complex_ for 5:1 and 10:1 molar ratios, respectively. These results showed that a 5:1 ratio is enough to have a high EE %.

#### NMR study

In order to elucidate the possible orientations of *trans*-Ner within the CD cavity, NMR spectroscopy was used. Because HP-β-CD is a heterogeneous mixture of isomers, NMR was conducted only with β-CD. ^1^H NMR and ROESY spectra were recorded for β-CD and β-CD/*trans*-Ner solutions in D_2_O. The induced shift (∆δ) which is the difference between chemical shifts of β-CD protons in the presence and absence of *trans*-Ner, was calculated as follows: Δδ = δ_(complex)_ – δ_(free)_. Positive and negative signs showed downfield and upfield displacement, respectively. The inclusion of the guest into the CD cavity is generally proved by the chemical shift variations of the guest or the CD. Particularly, a shielding of inner protons of the CD is induced after inclusion of a guest into the CD cavity while protons of the outside are generally unchanged [27]. It should be noted that in our study, the NMR spectra of *trans*-Ner alone in D_2_O could not be obtained due to its low aqueous solubility. The proton chemical shifts of β-CD and β-CD/*trans*-Ner inclusion complex solutions are shown in Table 3. As expected, the presence of *trans*-Ner shifted upfield the protons located inside the CD hydrophobic cavity (H_3_, H_5_ and H_6_) more effectively than those located at the surface (H_1_, H_2_ and H_4_). These observations confirmed that *trans*-Ner is included into the CD cavity without evidence for outside complexation. Besides, the higher upfield shift value of 0.12 ppm was assigned to H_5_ (Table 3). This probably means that the complexation of *trans*-Ner within the CD cavity involves hydrophobic interactions and that Ner penetrates the cavity from the wider side [28].

**Table 3:** ^1^H NMR chemical shifts (δ, ppm) for free β-CD and β-CD/*trans*-Ner inclusion complex solutions in D_2_O in the presence of 0.5 mM β-CD and 3.28 mM *trans*-Ner.

β-CD ^1^H	δ_(free)_ (ppm)	δ_(complex)_ (ppm)	∆ δ (ppm)

H_1_	5.11	5.08	−0.03
H_2_	3.70	3.67	−0.03
H_4_	3.62	3.61	−0.01
H_3_	4.00	3.93	−0.07
H_5_	3.89	3.77	−0.12
H_6_	3.91	3.83	−0.08

**Figure 4:**
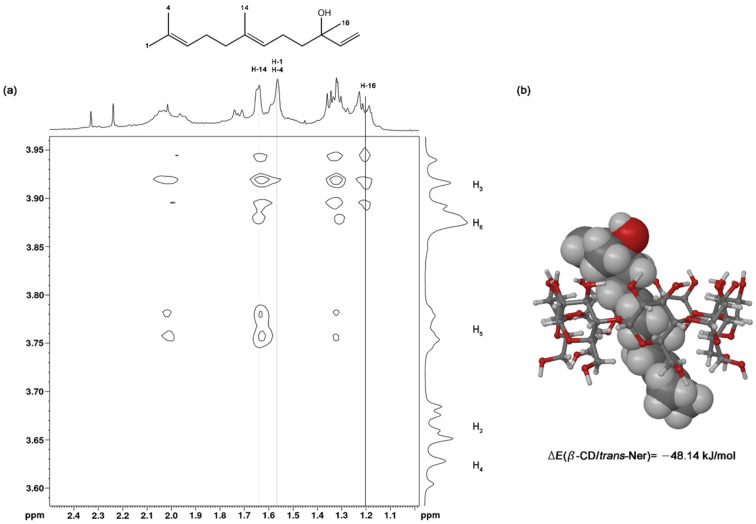
a) 2D ROESY spectrum of β-CD/*trans*-Ner inclusion complex in D_2_O and b) representation of the most stable inclusion complex conformer.

Furthermore, a ROESY experiment was performed to gain further insight into the dynamic structure of the β-CD/*trans*-Ner inclusion complex. The two-dimensional spectrum indicates a strong intermolecular cross-peaks between H_3_ and H_5_ protons of β-CD and the protons of *trans*-Ner’s methyl groups (Figure 4a). As can be seen in Figure 4a, the H-16 proton of *trans*-Ner interacted only with H_3_ proton of β-CD. Additionally, H-14 proton signals of *trans*-Ner showed intense cross-peaks with the inner CD protons, in particular with H_5_. However, H-1 and H-4 of *trans*-Ner did not interact with any of CD interior protons. Based on these data, we suggested that *trans*-Ner is included in the β-CD cavity where the hydroxy group is oriented towards the wider rim of β-CD and the methyl groups located at positions 1 and 4 are present outside the narrow side of CD.

#### Molecular modelling

The theoretical molecular modelling is useful to illustrate the most energetically favorable three-dimensional structure of the inclusion complex in solution. The inclusion complex conformer that presents the weakest relative binding energies (Δ*E*) (i.e., the most stable conformer) is represented in Figure 4b. The results showed that the formation of the β-CD/*trans*-Ner inclusion complex is an energetically favorable process and that the inclusion mode is coherent with the experimental NMR results.

#### Photodegradation studies of Ner

**Figure 5:**
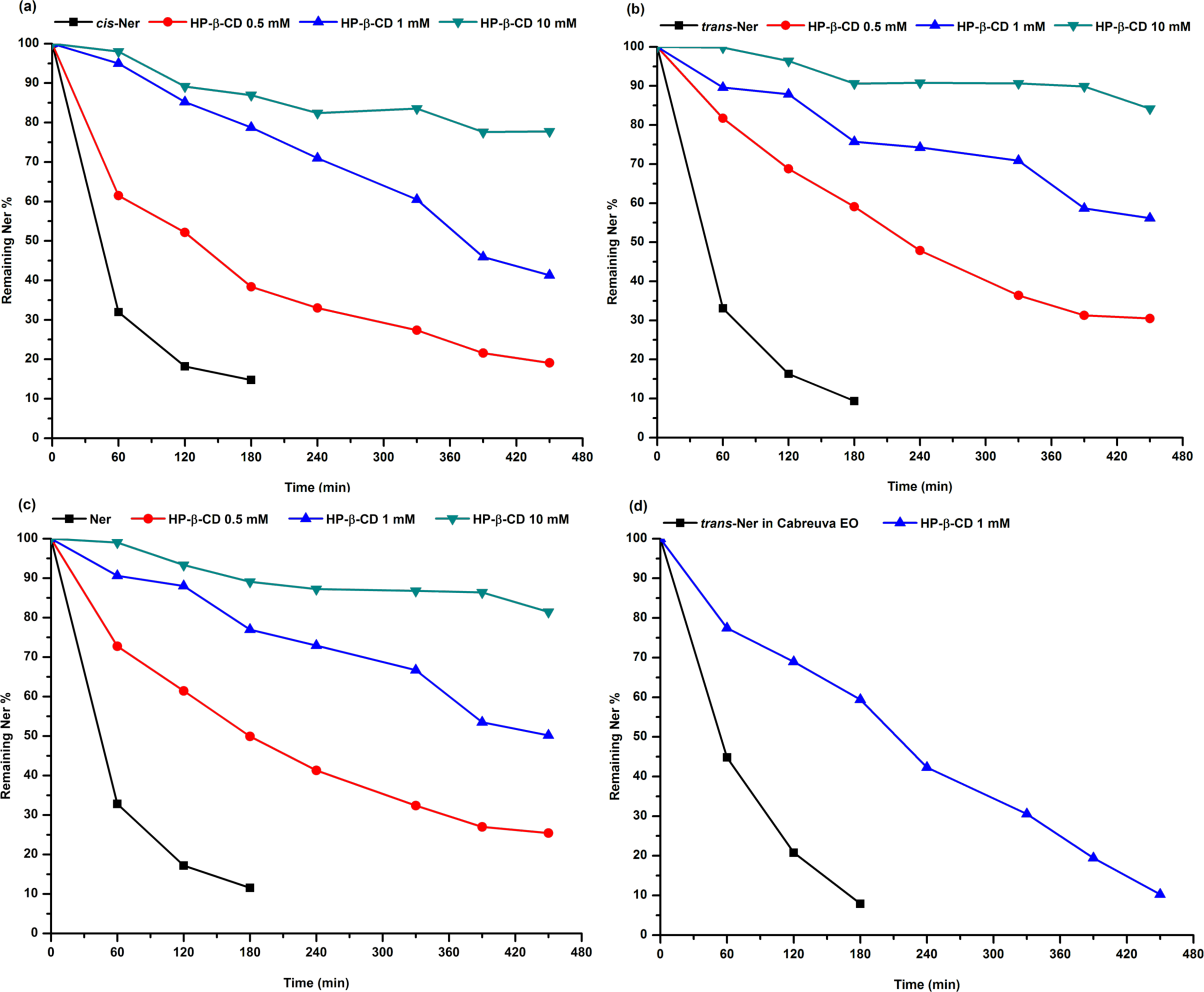
Photodegradation kinetics of *cis*-Ner (a), *trans*-Ner (b), the isomer mixture Ner (c) in the absence and presence of increasing concentrations of HP-β-CD (0.5, 1 and 10 mM) and of *trans*-Ner in cabreuva EO (d) in the absence and presence of 1 mM HP-β-CD under UV light irradiation.

To the best of our knowledge, no studies were performed on the photodegradation of Ner under UV-light. Nonetheless, we can assume that the presence of three C=C double bonds may indicate the susceptibility of Ner to be photodegradable [29]. Therefore, the photostability of Ner was carried out in aqueous solution, in the absence and the presence of different CDs (α-CD, β-CD, HP-β-CD and SBE-β-CD) after its irradiation with UVB light. The concentration of each isomer was determined by HPLC. The effect of HP-β-CD on the photostability of *trans*-Ner in the cabreuva EO was also assessed upon UVB exposure. The photodegradation reaction of all samples followed a first-order kinetics. First, a constant concentration of Ner (0.1 mM) was added to various concentrations of HP-β-CD (0.1, 1 and 10 mM). Figure 5 represents the percentage of *cis*, *trans*-Ner and the mixture remaining in the solution after irradiation in function of time. It can be observed that Ner was rapidly degraded in the absence of CD; after 120 min of UVB exposure, only about 17% of Ner remained in the solution (Figure 5a). However, its degradation was slowed down as the HP-β-CD concentration increased. For example, after 120 min of irradiation, about 60 and 90% of Ner was still remaining in the solution in the presence of 0.5 and 10 mM HP-β-CD, respectively. The inclusion of Ner into the CD cavity exhibited a protective effect against photodegradation. This effect was more important as the concentration of HP-β-CD increased, due to the larger amount of complexed Ner. Furthermore, the photostability of *trans*-Ner in the cabreuva EO was investigated with 1 mM HP-β-CD (Figure 5b). We can observe that *trans*-Ner in cabreuva EO was totally degraded after 180 min of irradiation; whereas the complexation of EO with HP-β-CD enhanced the stability of *trans*-Ner (only 40% of *trans*-Ner was degraded in HP-β-CD solution after 180 min, Figure 5b). The existing literature also showed the protective effect of CD towards other guests [13,30–33]. When comparing the photostability of *trans*-Ner in its pure form and in the EO, it can be noted that the degradation of *trans*-Ner alone (*K* = 0.0145 min^−1^) was slightly different than *trans*-Ner in EO (*K* = 0.0127 min^−1^). This finding could be explained by the presence of other chemical compounds in the EO that may be influence the degradation of *trans*-Ner [34].

Then, we compared the effect of different CDs at a fixed concentration (1 mM) on the photostability of Ner upon UVB irradiation. From Table 4, we can notice that in the absence of CD*, trans*-Ner is more rapidly degraded than *cis*-Ner (*K* = 0.0145 min^−1^ and *K* = 0.0121 min^−1^ for *trans* and *cis* isomers, respectively). The main reason behind its instability is that *trans*-Ner isomerizes to its *cis* form during irradiation [34–35]. However, the presence of CD improved the photostability of *trans*-Ner more efficiently compared to *cis*-Ner (Table 4). This could be explained by the fact that *trans*-Ner is better encapsulated in CD than *cis*-Ner (Table 1). For example, in the presence of SBE-β-CD, the *K* value of *trans*-Ner was 0.0007 min^−1^ while that of *cis*-Ner was 0.0012 min^−1^. This difference could be attributed to the difference in *K*_f_ values between the two isomers (*K*_f_ *_cis_*_-Ner_ = 9240 M^−1^ and *K*_f_ *_trans_*_-Ner_ = 13264 M^−1^). It is worth noting that the higher the *K*_f_ value, the higher the photoprotective effect of CD; thus, SBE-β-CD (*K*_f_ = 17768 M^−1^) > HP-β-CD (*K*_f_ = 10365 M^−1^) > β-CD (*K*_f_ = 1238 M^−1^) > α-CD (*K*_f_ = 418 M^−1^).

**Table 4:** Photodegradation rate constants (*K*, min^−1^, Equation 3) of CD/Ner inclusion complexes upon UVB irradiation.

K (min^−1^)	*cis*-Ner	*trans*-Ner	Ner

No CD	0.0121	0.0145	0.0134
α-CD	0.0051	0.0051	0.0051
β-CD	0.0019	0.0010	0.0013
HP-β-CD	0.0017	0.0012	0.0014
SBE- β-CD	0.0012	0.0007	0.0009

## Conclusion

In this study, inclusion complexes of Ner (*cis*, *trans* and the isomer mixture) with seven CDs were successfully formed in aqueous solution. All inclusion complexes exhibited an 1:1 (CD:Ner) stoichiometry, with *K*_f_ values of CD/*trans*-Ner superior to those of CD/*cis*-Ner. The solubility of Ner and of the cabreuva EO (containing 64.54% of *trans*-Ner) was greatly enhanced in the presence of CDs. Moreover, solid HP-β-CD/Ner inclusion complexes were obtained with a high encapsulation efficiency (EE %). Structural characterization by NMR and molecular modelling confirmed once again the formation of the inclusion complex between *trans*-Ner and β-CD. The most stable structure was obtained for the hydroxy group oriented towards the wide rim of the CD. Finally, the photostability of Ner and of *trans*-Ner in cabreuva EO was remarkably improved upon CD complexation, thereby proving the protective effect of CD. From the above results, we could conclude that CDs are effective encapsulating agents for Ner, able to enhance its solubility and photostability.

## Experimental

### Materials

Nerolidol (98%, mixture of *cis* (40%) and *trans* (60%) isomers), *trans*-nerolidol (≥85%, analytical standard) and thymol (>99%) were purchased from Sigma-Aldrich (Missouri, United States). Cabreuva (*Myrocarpus fastigiatus*) essential oil (64.54% of *trans*-Ner) was purchased from Herbes et Traditions (Comines, France). Methylorange (MO) was provided by Acros Organics (Massachusetts, United States). α-CD, β-CD, HP-β-CD (DS = 5.6) and RAMEB (DS = 12.6) were purchased from Wacker-Chemie (Lyon, France). CRYSMEB (DS = 4.9) was provided from Roquette Frères (Lestrem, France). SBE-β-CD (DS = 6.5, Captisol^®^) was donated by Ligand Pharmaceuticals Incorporated (La Jolla, USA). Methanol HPLC-grade was purchased from VWR Chemicals (Atlanta, United States).

### Methods

#### HPLC assay

Chromatographic analysis was performed with a Waters 600E multisolvent delivery system HPLC. The method development for estimation of Ner quantity in CD was carried out using the analytical column Xterra RP C18 (150 × 4.6 mm, 5 µm). The column temperature was maintained at 25 °C. The mobile phase consisted of methanol/water (70:30 v/v). The flow rate was set at 1 mL/min. The injection volume was 20 µL. The wavelength of UV detector was set at 212 nm. Stock solutions of Ner (1 mg/mL) and thymol (1 mg/mL) were prepared in methanol. Then, diluted concentrations of Ner ranged from 5 to 250 µg/mL and 100 µg/mL thymol, used as internal standard, were prepared in methanol. For HPLC measurement, 100 µL of each sample was added to thymol (100 µL) and methanol (200 µL). The mixture was then sonicated at 4 °C and centrifuged at 15000 rpm at 4 °C. The HPLC method was validated in terms of linearity, repeatability and limit of detection.

### Formation constants determination

#### UV–visible spectroscopy

The UV–visible competition method described by Landy et al. [23] was used to determine the formation constants (*K*_f_) of HP-β-CD/Ner inclusion complexes. Methylorange (MO), an azo dye, was used as a competitor. First, the *K*_f_ of the HP-β-CD/MO inclusion complex was calculated by a direct titration method. Then, a spectral displacement method was performed by adding Ner to a solution containing constant concentrations of HP-β-CD and MO. The expulsion of MO from HP-β-CD cavity induced an absorbance increment. The spectrum of MO solution (0.1 mM) was recorded between 520 and 530 nm. In this range of wavelengths, there was an optimal difference in absorbance between the free and encapsulated forms of MO. The HP-β-CD/Ner *K*_f_ values were calculated by an algorithmic treatment to minimize the difference between the experimental and theoretical values of the peak area. The *K*_f_ calculation is based on 1:1 stoichiometry for HP-β-CD/MO and HP-β-CD/Ner. All measurements were done using a UV–visible dual-beam spectrophotometer with a 1 cm thick quartz cuvette (Perkin Elmer Lambda 2S).

#### Phase solubility studies

Phase solubility studies were performed according to Higuchi and Connors [36]. Excess amounts of Ner were added to 2 mL of CD solution (α-CD, RAMEB, CRYSMEB, HP-β-CD or SBE-β-CD) at concentrations varying from 0 to 10 mM, except for γ- and β-CD where lower concentrations were used (0–1 mM). The mixtures were shaken for 24 h at 25 °C. After the equilibrium was reached, the solution was filtered through a 0.45 μm filter. The concentrations of *cis* and *trans*-Ner were determined using the HPLC method described above. The solubility of Ner was plotted against CD concentration. Formation constant (*K*_f_) values of CD:Ner inclusion complexes were calculated from phase-solubility diagrams using the following equation:

[1]
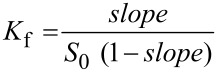


where *S*_0_ is the intrinsic solubility of Ner in aqueous solution without CD.

The solubilizing effect of CD was also determined by calculating complexation efficiency (*CE*) parameter which is the concentration ratio between CD in a complex and free CD:

[2]
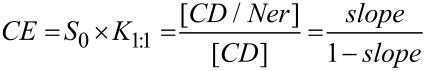


### Total organic carbon (TOC) analysis

Phase solubility studies of cabreuva EO with HP-β-CD were investigated by TOC analysis using a Shimadzu TOC-VCSH analyzer. It is based on the production of carbon dioxide (CO_2_) following oxidation of organic compounds. CO_2_ is then detected using a high-sensitivity infrared gas analyzer (NDIR). First, TOC was measured for the CD solution (TOC_CD_). Then, the amount of EO in the filtrate was calculated using the following equation: TOC_EO_ = TOC_T_ − TOC_CD_, where TOC_T_ is the TOC value obtained for the filtrate by the TOC analyzer. Results were reported in g/L of organic carbon. The solubility of EO was determined from standard curves constructed with known EO concentrations.

### Preparation of the solid inclusion complex by freeze-drying

Ner (10 mM) was added to different HP-β-CD (50 and 100 mM) aqueous solutions. Solutions were stirred at 300 rpm for 24 h at 25 °C. After equilibrium was reached, the suspensions were filtered, frozen at −20 °C for 24 h and lyophilized. The lyophilization process was carried out at −85 °C and 0 Pa in a Christ Alpha 2-4 LD Freeze dryer until all moisture had been sublimated.

### Encapsulation efficiency

Encapsulation efficiency (EE %) of Ner as a mixture (10 mM) into HP-β-CD (50 and 100 mM CD) was carried out by dissolving 10 mg of solid inclusion complexes in ethanol (10 mL). An aliquot of each sample was taken and analyzed by the HPLC method described above.

The EE % was calculated as follows:

[4]
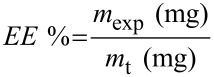


where *m*_exp_ and *m*_t_ are the experimental and theoretical (amount of Ner initially added to prepare the solid inclusion complex) quantities of Ner in the solid inclusion complexes, respectively.

### Molecular modelling studies

Molecular mechanics simulation was done using the Macromodel-MMFFs force field in the presence of water (GB/SA implicit model) as implemented in the Schrodinger software (release 2014). The CD hosts were based on a non-distorted monomeric β-CD with C7 symmetry. *trans*-Ner were constructed manually. Fifty conformations of *trans*-Ner/CD complexes were selected (Monte Carlo conformational searches: Mixed torsional/low mode sampling, 10000 structures generated, FMNR conjugate gradient minimization, convergence fixed to 0.01 kJ Å^−1^ mol^−1^). The total energy difference (Δ*E*, kJ/mol) between the inclusion complex and the sum of their individual components in their optimized fundamental states was calculated (Δ*E* = *E*_CD/Ner_ – (*E*_CD_ + *E*_Ner_)) and used as the theoretical parameter to evaluate the complexation energy of the inclusion complex.

### NMR spectroscopy

NMR experiments were realized for β-CD/*trans*-Ner inclusion complex solution. ^1^H NMR spectra were acquired on a 400 MHz Bruker Avance III spectrometer at room temperature equipped with a multinuclear z-gradient PABBO probe head capable of producing magnetic field pulse gradients in the z-direction of 48.15 G·cm^−1^. *trans*-Ner and β-CD were dissolved in 99.98% D_2_O. Two-dimensional (2D) ROESY off resonance spectra were acquired at 25 °C with presaturation of the residual water resonance and a mixing (spin-lock) time of 600 ms, using the States-TPPI method with a 2048 K time domain in F2 and 512 experiments in F1. Sixty-four scans were recorded to obtain a good resolution 2D ROESY spectra.

### Photostability study

The Ner photodegradation experiment was realized under an artificial UV source (UVB, 310 nm) in a 100 mL quartz reactor, with continuous stirring, using a Multirays apparatus (Heliosquartz, Italy). The photostability of Ner, cabreuva EO and their inclusion complexes with CDs was investigated under UVB irradiation as a function of time.

Ner (0.1 mM) was added to solutions of various HP-β-CD concentrations (0.5, 1 and 10 mM) and to 1 mM solutions of α-, β- and SBE-β-CD. Cabreuva EO (1.27 µL) was also added to 1 mM HP-β-CD aqueous solution. Ner and cabreuva EO aqueous solutions without CD were also prepared. Irradiated aliquots were withdrawn every 60 min, over 8 h of total irradiation and the remaining Ner concentration was then determined by HPLC.

The percentage of Ner remaining was calculated as follows:

[5]
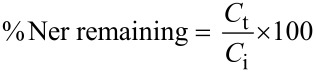


where *C*_t_ is Ner concentration in solution after irradiation at time t and *C*_i_ is Ner concentration before irradiation.

The photodegradation constant (*K*, min^−1^) was also calculated using the following equation:

[3]
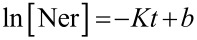


## Supporting Information

File 1 ^1^H NMR spectra.
