# Supplementary material for: The effect of cyclodextrin complexation on the solubility and photostability of nerolidol as pure compound and as main constituent of cabreuva essential oil
Source: Beilstein J Org Chem. 2017 May 5;13:835–44. doi: 10.3762/bjoc.13.84 (PMC5433144; doi:10.3762/bjoc.13.84)
Supplement: File 1 — 1H NMR spectra. [file Beilstein_J_Org_Chem-13-835-s001.pdf]

Supporting Information

for

The effect of cyclodextrin complexation on the solubility and photostability of nerolidol as pure compound and as main constituent of cabreuva essential oil

Joyce Azzi[1,2], Pierre-Edouard Danjou[2], David Landy[2], Steven Ruellan[2], Lizette Auezova[1], Hélène Greige-Gerges[1] and Sophie Fourmentin*[2]

Address: [1]Bioactive Molecules Research Group, Doctoral School of Sciences and Technologies, Faculty of Sciences, Jdaidet El-Matn, Lebanese University, Lebanon and [2]Unité de Chimie Environnementale et Interactions sur le Vivant (UCEIV, EA 4492), SFR Condorcet FR CNRS 3417, ULCO, F-59140 Dunkerque, France.

Email: Sophie Fourmentin - lamotte@univ-littoral.fr

*Corresponding author

^{1}H NMR spectra

(a)

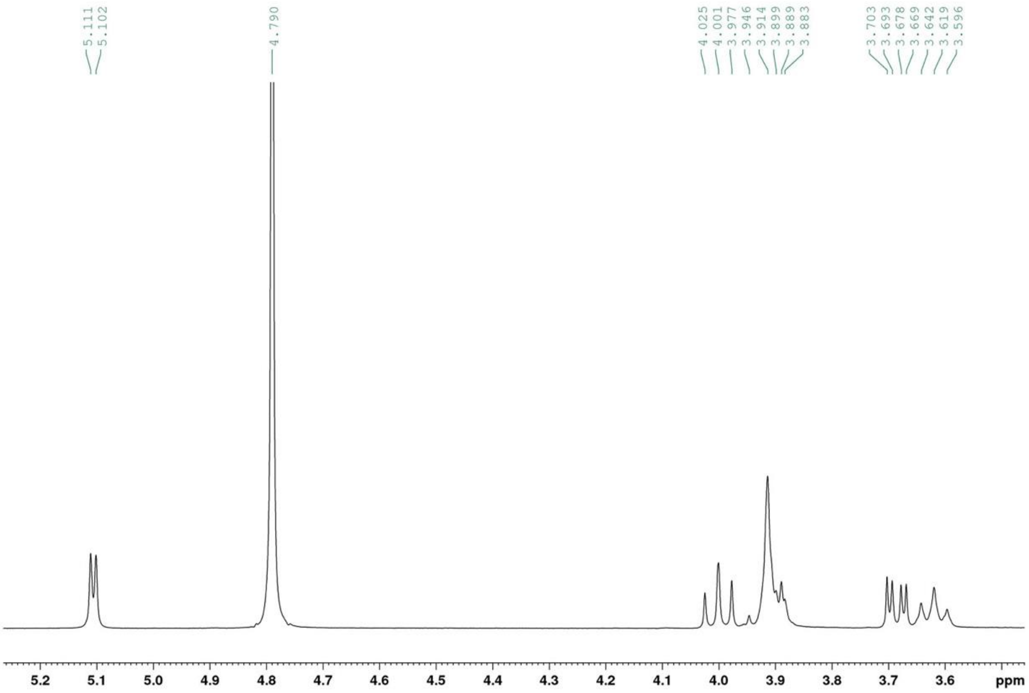

(b)

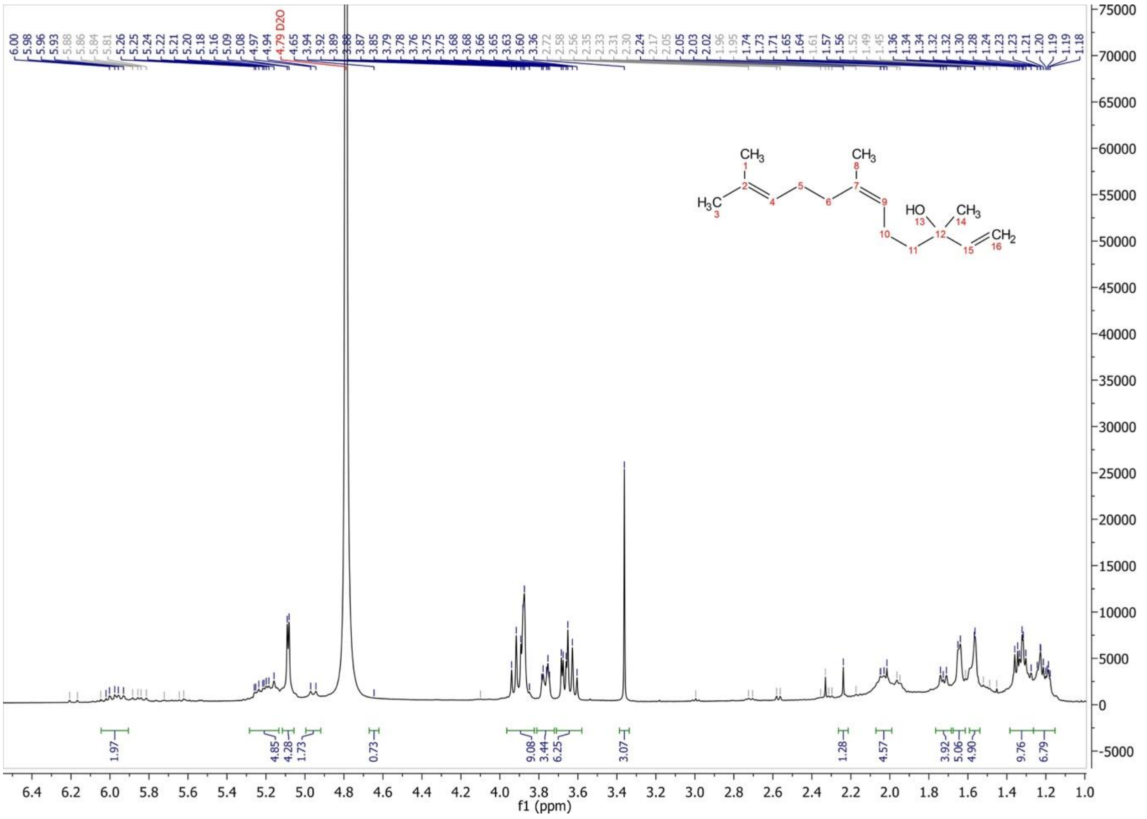

Figure 1: 1H RMN spectra for β-CD (a) and β-CD/trans-Ner inclusion complex (b) solutions in D_2O.
